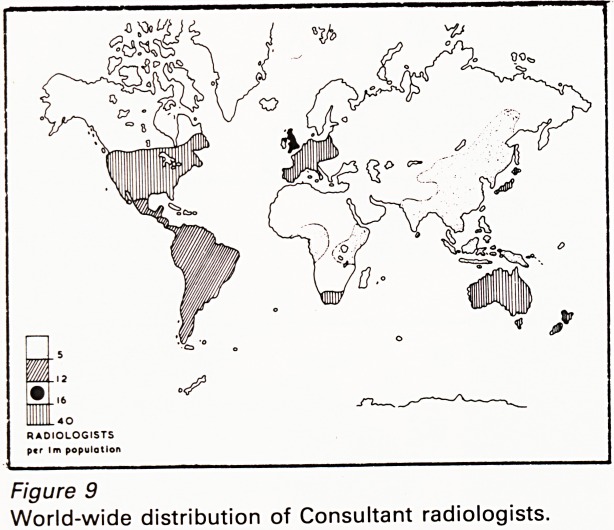# Images and Measurements in Clinical Radiology

**Published:** 1982

**Authors:** E. Rhys Davies

**Affiliations:** Professor of Radiodiagnosis, University of Bristol


### Images and Measurements in Clinical Radiology

### (Based on an Inaugural Lecture given at the Medical School on 25th February 1982)

**E. Rhys Davies**
Professor of Radiodiagnosis,
University of Bristol

Academic radiology got off to a slow start. The first Professor of Diagnostic Radiology was appointed in 1917 at the Karolinska Hospital in Stockholm, and the second Professor was not appointed until 1931, in Uppsala. In this country, the first full-time Professor of Diagnostic Radiology to be appointed to an undergraduate teaching hospital was in the University of Wales at Cardiff in 1965, and it gives me a vicarious pleasure to record that he is both Welsh and came from Bristol. Previously the University of Leeds has a part-time professorial appointment which subsequently became vacant, and the Royal Postgraduate Medical School had appointed a Professor in 1960. Having repatriated a Professor to Wales in 1965, the University of Bristol appointed its own Professor in 1966 (Bull, 1972). Since that time no fewer than five radiologists who underwent part of their training in Bristol have been appointed to Chairs in Radiodiagnosis in four different continents.

In contrast clinical radiology got off to an explosive start immediately after X-rays were discovered by Roentgen in 1896, arguably one of the few major products of pure research that have been applicable to clinical medicine. It is said that behind every famous man there is a slightly astonished wife, none more so perhaps than Frau Roentgen, who can hardly have imagined that the radiograph of her left hand which launched diagnostic radiology, would become a radiological heirloom. The first radiographs to be produced were noteworthy on the one hand for their ability to record a recognisable image of the region being studied, and on the other hand for the rather indistinct outline and poor definition of the contents of the image. There is a striking contrast between these early images and the present ability to record fine trabecular tracery with a sharp definition of edges and margins. Other methods of producing images have been explored, and the most striking of these is another example of pure research being applied to the clinical field. In

*Figure 1*

(a) CT upper abdomen

(b) Line diagram.
A = Aorta; L = Liver; Sp. = Spleen;
St. = Stomach with contrast.

Note

1. High density of vertebra, contrast-filled stomach and retrocrural lymph node.
2. Intermediate density of liver, spleen, and aorta.
3. Low density of subcutaneous and retroperitoneal fat.
4. Least density of air in the right costophrenic sulcus.

computed tomography (CT) the X-ray absorption coefficient is calculated for each of a grid of elements measuring 64 x 64, or 128 x 128 etc., and is then allocated a colour code or a grey scale before the final cross sectional image is written out, by computer (Figures 1a & 1b).

The conventional image of radiology is that of an expanding speciality, and the expansion can be measured by considering both the increases in the number of radiologists throughout the country and also the increase in the area of radiographic film being used each year (Figures 2a & 2b). Despite the increase in the numbers of radiologists, there is a shortfall in the total number and despite the increase in the amount of film the nature of the investigations responsible for this is concealed. For example, ultrasound and computed tomography absorb a great deal of radiologists' time, although they use relatively little film. Finally clinicians expect frequent discussions about clinical management with radiologists, making new and very important demands on their time.

However, merely recording an image is no longer enough to satisfy all the clinical requirements. It is appropriate to regard each radiograph as a unique record of anatomical events at a precise time. Thus a chest radiograph can show a small pulmonary granuloma very accurately. Also it is capable of recording the pulmonary tuberculous cavity that supervenes when the granuloma breaks down. In this way serial radiographs record change and can be regarded as a dynamic sequence with a long time base. CT is used in a similar way. Transverse sections through the trunk give a new dimension to anatomical imaging, and serial images can show regression of tumour as it responds to treatment (Figures 3a & 3b). Despite the unique quality of these anatomical images, it is dangerous to draw conclusions about *function* from them. The problem posed by the need to measure function can be tackled in a number of ways.

*Figure 2*
(a) Annual percentage expansion of the Consultant grade in England and Wales, 1967-78.
(b) Area of radiographic film consumed in England and Wales 1967-77.
These graphs are based on DHSS statistics.

### MEASURING A SPECIFIC FUNCTIONAL PARAMETER

Firstly by using ultrasound techniques to measure the velocity of blood in arteries. The velocity varies cyclically from systole to diastole. The shape of this cyclical wave form is influenced by arterial stenosis, and comparison of its shape at different levels in a limb help to determine the significance of atheroma and can be used to help select patients for arteriography. The latter is still an essential preliminary to planning surgical approach. The development of ultrasound techniques such as this has become a major contribution of the vascular laboratory developed by the Department of Medical Physics at Bristol. Not only has it brought international renown to the Department, it has established an important liaison between basic science and clinical medicine – one of many fruitful liaisons that are particularly relevant to the diagnostic radiologist.

Secondly isotopic techniques can be used to measure perfusion. By placing the detecting crystal of a gamma camera over a region of the body, and making an intravenous injection of a radioactive bolus, it is possible to record the distribution of radioactivity in the field of interest at intervals as frequent as one per second. This record can be played as a cinefilm or all the

*Figure 3*
CT upper abdomen
(a) Before treatment, showing retroperitoneal mass obliterating the outlines of aorta and inferior vena cava.
(b) After radiotherapy, showing reduction in the size of the mass. The outlines of the aorta and vena cava are now distinct.

images can be summated to form a totalised image. The intensity of the radioactivity is allocated to a colour from a pre-selected range. The data can be displayed as activity/time curves over a selected region of interest, and comparison between these curves can be used to detect localised ischaemia. The technique is an excellent discriminator between acute torsion of the testis needing urgent surgery from other acute scrotal conditions which are managed conservatively (Thomas et al., 1980).

### DEMONSTRATION OF AN ABNORMAL PHENOMENON – DUODENOGASTRIC REFLUX

The original technique for demonstrating duodenogastric reflux involved duodenal intubation, but in recent years an alternative technique has been developed, not involving intubation. A radiopharmaceutical that is excreted in the bile is injected intravenously so that the duodenal contents are made radioactive within some half hour of injection. Normally this radioactivity only spreads distally into the gut. The presence of proximal radioactivity is always abnormal and its intensity is used to measure the degree of reflux.

### EJECTION FRACTION OF A CONTRACTING ORGAN

Let us stay with the gall bladder, which we now know can be identified by either radiographic or isotopic techniques. Radiographs show how well the normal gall bladder contracts in response to a fat meal and how poorly it contracts under the same stimulus in some patients with coeliac disease. Comparisons are made between patients or groups of patients, by measuring the degree of contraction, and this can be done in two ways.

(a) Assume that the gall bladder is symmetrical about its long axis, like a lemon, and imagine it being cut into an infinite number of thin circular slices. If the diameter and height of each section are known, its volume can be calculated, and the volume of the whole is the sum of the volumes of the parts. This principle can be applied to measuring tracings of the gall bladder made from radiographs taken before and after contraction.

(b) The second technique is less tedious and more elegant. If a balloon is immersed in a water bath placed under a device for measuring radioactivity, it can be inflated with known volumes of a radioactive solution, and the recorded countrate compared with the known volume. The two measurements, plotted against each other will lead to a straight line passing through the origin. Thus if radioactivity is distributed evenly within the gall bladder, and the gall bladder centre does not move away significantly from the detecting crystal, changes in countrate are proportional to changes in volume. Comparison of these techniques in measuring gall bladder contraction shows a good correlation (Figure 4). During in vivo studies, fluctuating background activity is represented by an intercept on the ordinate. Such back-

*Figure 4*
Contraction of the gall bladder determined by change in radioactive counts per minute (ordinates) and volume calculated from radiographic measurements (abscissae). There is a good correlation and the intercept on the ordinate corresponds to background activity. If allowance can be made for this background, change in countrate can be used to measure percentage change in volume of a contracting organ, whether it is the gall bladder or the left ventricle (Chapple et al., 1975).

ground activity often makes it difficult to define precisely the end point between the radioactive target and the less radioactive background.

### LEFT VENTRICULAR EJECTION FRACTION

The inherent dangers of making inferences about function from purely anatomical studies can be abolished by reducing the timescale between serial images to a fraction of a second, as in cinecardiography, which is used to demonstrate function of the left ventricle. The technique requires careful percutaneous catheterisation of the left ventricle and carries some hazard even in experienced hands. The end diastolic and end systolic points can be identified, and the 'lemon technique' can be used to measure the ejection fraction. By the same token as in gall bladder studies, isotopic techniques can be used for this purpose also. They have the advantage that it is necessary only to make intravenous injections in order to label the red blood cells with $^{99}$Technetium$^{m}$. By use of a computer programme triggered through an electrocardio-graph, it is possible to summate radioactivity over a large number of cardiac cycles in order to give one cyclical record with an appropriate target to background ratio. It is still difficult to define the end point of the ventricle accurately, but it has been possible to devise a technique that will enable this to be done by computer (Jackson et al., 1982). The technique can be used to demonstrate contraction and measure the ejection fraction. Among its many uses are the detection of akinesia, dykinesia, ventricular aneurysm; measurement of the severity of ventricular dysfunction; measurement of the response of ventricular function to appropriate treatment; helping to select patients suitable for cardiac surgery etc.

### MEAN TRANSIT TIME

After exploring the possibilities for measuring physiological events, and recording pathological events, it is inevitable that we should arrive at the theoretical ultimate – a valuable measurement that bears no relationship to any physiological parameter. Renography is a well-established technique for recording the transit of radioactivity through the kidneys, and its retention and excretion by the kidneys. The familiar curves have been used empirically and successfully to help the management of renal disease. They are a complex summation of several events which cannot be separated and an important step was taken when mathematicians proposed an analysis of the curves that would suggest a theoretical final curve based on a standardised simplified model of the kidney. If a small volume of radioactivity could be introduced instantaneously into a single tube, and allowed to pass along it until it disappeared equally instantaneously, it would be possible to record these events with a detecting crystal (Figure 5). In practice the input curve is exponential and the renal retention function (or renogram) is the summation of the retention fuctions of each of its nephrons, which are unlikely to be uniform (Figure 6). Given the exponential input function and the crude retention function or renogram, it is possible, by a mathematical manipulation or deconvolution, to draw a curve which would have occurred in the kidney being studied, if the input function had in fact been instantaneous (Figure 7) (McAlister, 1979).

This curve is best defined by taking its centroid and recording the time that corresponds to the intercept of the centroid on the abscissa. This is the so-called *mean transit time* and it can be applied to the management of:

(a) *Hydronephrosis*

The diagnosis of hydronephrosis is made

*Figure 5*

Mean Transit Time. Imagine a renal model consisting of a single tube placed under a detecting crystal so that a bolus of radioactivity can be monitored as it passes along the tube. The following features will be recorded:
Above:
input trace – showing an instantaneous input
Middle:
transit spectrum – showing instantaneous disappearance
Below:
retention function – a square wave corresponding to the duration of radioactivity in the tube

*Figure 6*

In practice the renogram has the following parameters:
Above:
the input function is exponential with re-circulation peaks
Middle:
the transit spectrum is spread because the individual nephron transits differ from each other
Below:
the retention function has the characteristic shape of a standard renogram

*Figure 7*
In deconvolution analysis of the curves in Figure 6, it is assumed that the input function is instantaneous (above) that the transit spectrum is the same (middle) and the retention function is calculated and plotted (below). This function is best described by the intercept of its centroid on the abscissa, called the mean transit time (after McAlister, 1979).

readily by excretion urography which enables detection of delayed transit through the renal parenchyma and delayed drainage from the renal pelvis. The mean transit time is a useful way of recording the severity of both these abnormalities and it has been proposed that it is a useful way of selecting patients in need of urgent operation as well as following post-operative progress (Figure 8).

(b) *Renal Colic*
The conservative treatment of renal colic poses numerous problems for the diagnostic radiologist. Excretion urography is of course the established initial investigation but there are several objections to its frequent use in following-up the long-term effects of stones on renal function. These objections do not apply to isotopic investigations; and the mean transit time is already promising for investigating and following-up renal colic.

### THE SELECTION OF APPROPRIATE TESTS

The ability to measure so many phenomena poses major problems of choice between different methods of investigation. The line of least resistance might well be to carry out all the tests that bear on the problem in the hope of making sufficient unbiased observations to arrive at a correct diagnosis. It is analogous to covering a canvas with different colours indiscriminately, in the hope that a portrait will emerge. It is an absurd way to try to solve a problem, and is far removed from the scientific approach that it sometimes purports to be (Sherwood, 1978). One of the more important radiological themes in recent years has been the need to devise a diagnostic pathway that is a simple and economic way of achieving the critical information being sought. The involvement of radiologists in devising such a pathway is, next to the technological advances I have mentioned, the most important single change that the discipline has witnessed in recent decades.

Sometimes the choice is simple, as for example in the application of ultrasound techniques to the gravid uterus. There is no radiation or other known hazard, and with the advent of real time instruments, it has become possible to solve acute problems with a high degree of accuracy, and to monitor the safe progress of a normal pregnancy.

On the other hand several techniques might be available to investigate different aspects of the same problem. Thus following hepatic injury, the simplest way of detecting an intrahepatic haematoma may well be the use of ultrasound, and the alternative application of isotopic tests or

*Figure 8*
Left Hydronephrosis
(a) Excretion urogram.
(b) Standard renogram (above), deconvolution curve and mean transit time (below). The transit time over the left kidney is markedly prolonged.

computed tomography may be influenced by cost and availability. However, if the clinical problem is to determine the cause of persisting haemobilia following liver trauma, the critical investigation is an arteriogram which will illustrate the point of leakage. These and other considerations will apply also to the investigation of suspected liver abscess, which could call upon ultrasound, gallium scanning, colloid scanning or computed tomography.

The whole concept of the 'critical pathway' implies being able to select an investigation that will help to confirm a provisional or 'best' diagnosis. On the other hand, it may refute that diagnosis and the whole sequence may need to be rethought.

It is no mischance that I have used the word 'clinical' in order to define the kind of radiology that I have been describing. Unfortunately the word is established firmly among the misused and cliché words and recently even the cricket correspondent of *The Times* described an innings by a well-known cricketer as 'ruthless and clinical'. Presumably he meant that he was batting coldly and dispassionately (Howard, 1977). The word is, of course, derived from the Greek word 'clinos' – a bed, and although radiology, as I have indicated earlier, is not the oldest profession in the world, it is in the sense of radiology being applicable to the bedside and to the decisions that are taken there that I use the word 'clinical'. The range of skills available to the radiologist in this application is constantly increasing, and it is clear that medical physics, mathematics, computer development, and radiopharmacy have much to offer the practice of clinical medicine. Many interfaces have been established between them already, and radiology and radiologists increasingly find themselves at one of these interfaces. The profitable liaison that has been established in this University between science on the one hand, and radiology and other clinical disciplines on the other hand, is of course not unique, but it is not universal either, and it does point the way very firmly to future developments in all our discipline.

The potential of clinical radiology is limitless, but time and people are not. It would be inappropriate for me to conclude without giving brief considerations to the following questions.

### HOW MANY PEOPLE ARE NEEDED?

There is no universally accepted method of *measuring* the number of radiologists needed, and I have to rely on an *image,* that of the number of radiologists there are world-wide (Figure 9). The distribution falls into three well-defined groups, and Great Britain occupies an intermediate position between the best of the under-developed nations and the worst of the industrialised nations. In short, there are far too few radiologists in Great Britain, despite the fact that the number of X-ray investigations being generated per annum – 400 per 1,000 population (Kendall et al., 1980) – is lower than that in other industrial countries.

### HOW CAN THEY BE MADE AVAILABLE

Radiological training is essentially postgraduate and beyond this discussion, but it is evident that no medical education is complete without an awareness of radiology. Nearly everyone who is admitted to hospital is radiographed and

*Figure 9*
World-wide distribution of Consultant radiologists.

inevitably radiology pervades medical education, as it does medical practice. The object of basic medical education should be to provide a basis for future vocational training (General Medical Council, 1967). Radiology can be involved in this in three ways.

1. As a vehicle for training. Radiology is available for helping to make anatomical, physiological and scientific considerations relevant to medicine.
2. Medical students should be exposed to the clinical uses and applications of radiology through the medium of seminars and discussions, but not exposed to systematic teaching.
3. There should be adequate general preparation for individuals to pursue a career in radiology.

In considering that preparation, I venture to draw your attention to a statement made before this University in 1929.

> 'The most important thing about education is appetite.'
>
> (Churchill, 1929)

Our aim is to give the student an appetite for diagnostic radiology.

I hope, Mr. Dean, that I have not broken the restrictions set out in the first half of my final quotation, as my intention was only to follow the precept of its second half.

> 'The mind does not need filling up like a vessel, merely kindling like fuel.'
>
> (Plutarch)

### REFERENCES

BULL, J. W. D., 1970. The Place of Radiodiagnosis in Medical Education: Methods Proceedings of the Royal Society of Medicine, 63, 835-837.

CHAPPLE, M. J., NOLAN, D. J., LOW BEER, T. S., DAVIES, E. R., 1975. Gall Bladder emptying measured by a radioisotopic method, *British Journal of Radiology,* **48**, 19-22.

CHURCHILL, W. S., 1929. Address to Bristol University.

HOWARD, P., 1977. New Words for Old. Hamish Hamilton, London, 13-15.

JACKSON, P. C., ALLEN-NARKER, R., DAVIES, E. R., REES, J. R., WILDE, P., WATT, I., 1982. The assessment of an edge detection algorithm in determining left ventricular ejection fraction using radionuclide multiple gated acquisition and contrast ventriculography. *European Journal of Nuclear Medicine* in the Press.

KENDALL, G. M., DARBY, S. C., HARRIES, S. V., RAE, S., 1980. A Frequency Survey of Examinations carried out in NHS Hospitals in Great Britain in 1977 for Diagnostic Purposes, National Radiological Protection Board.

McALISTER, J. M., 1979. Radionuclide Techniques in Medicine, Cambridge University Press, pp.155-157.

PLUTARCH de AUDIENDO, quoted by Pickering G. W., 1978. Quest for Excellence in Medical Education, Nuffield Provincial Hospitals Trust.

SHERWOOD, T., 1978. Science in Radiology, *Lancet,* **1**, 594-595.

THOMAS, W. E. G., COOKE, P. H. DAVIES, E. R., JACKSON, P. C. WILLIAMSON, R. C. N., 1981. Dynamic radionuclide scanning of the testis in acute scrotal conditions, *British Journal of Surgery,* **68**, 621-624.

## Figures and Tables

**Figure 1 f1:**
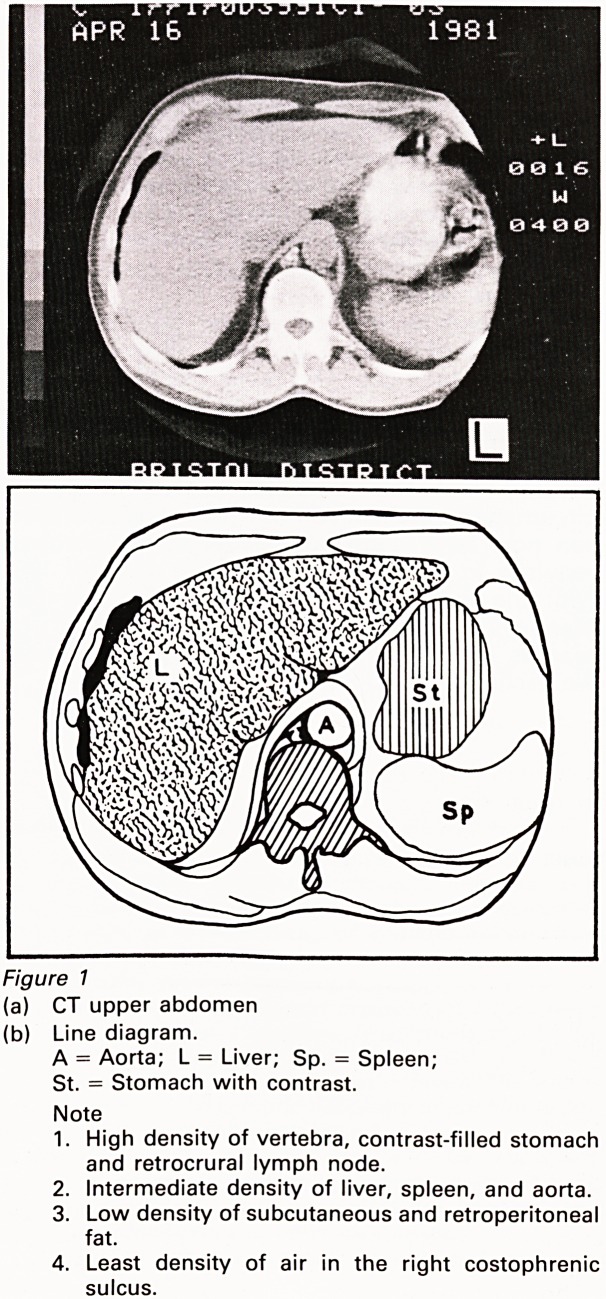


**Figure 2 f2:**
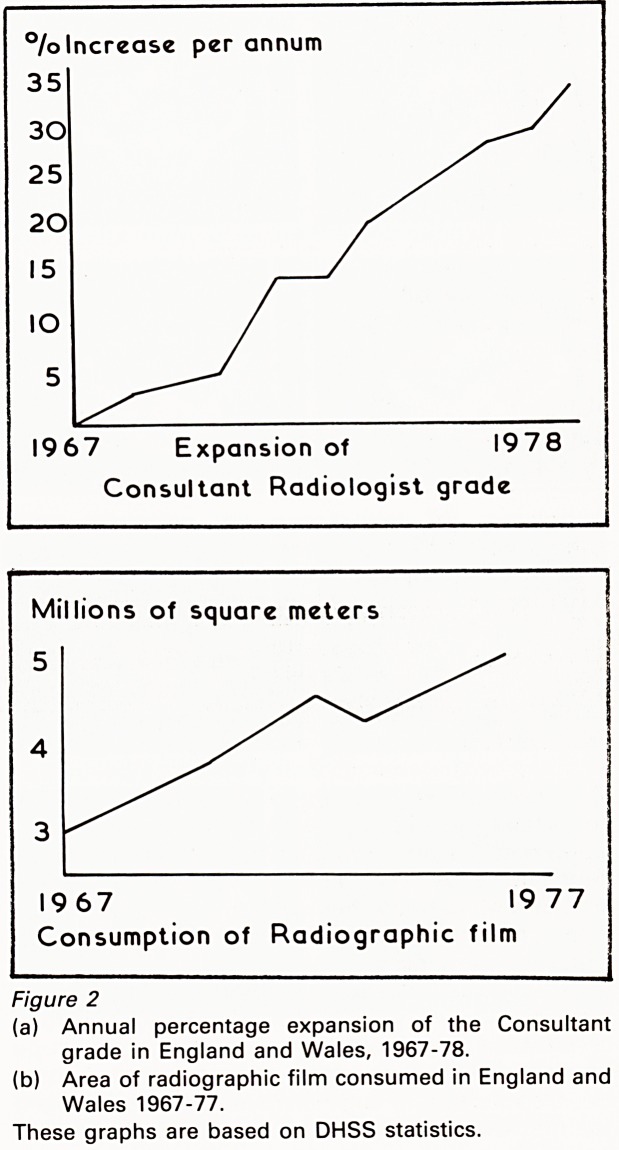


**Figure 3 f3:**
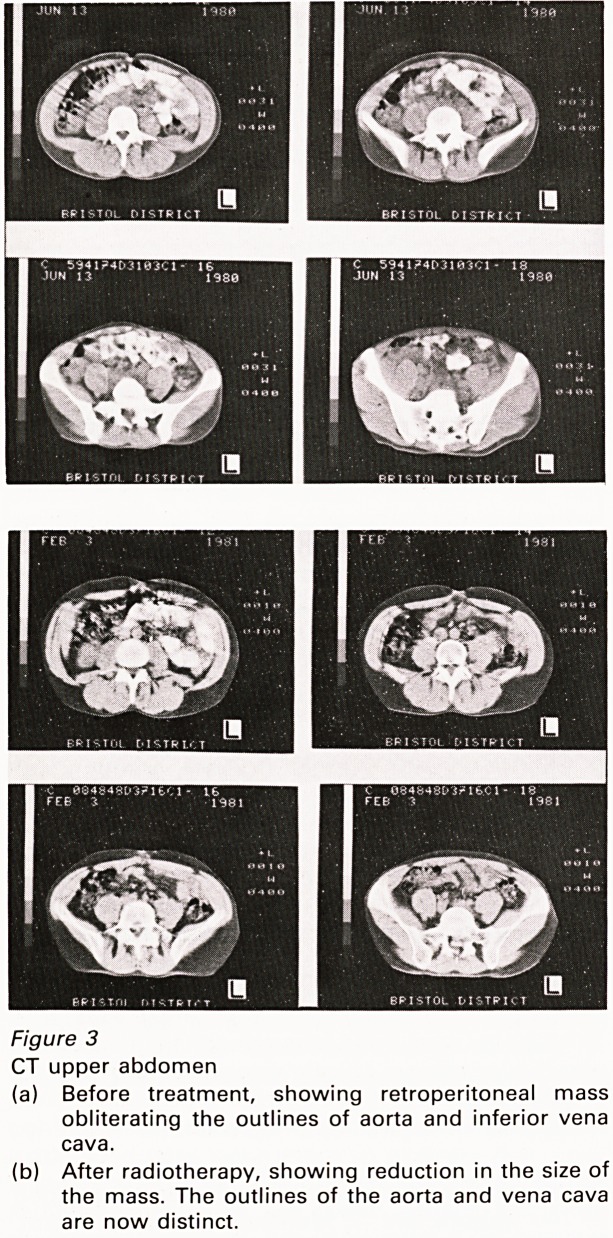


**Figure 4 f4:**
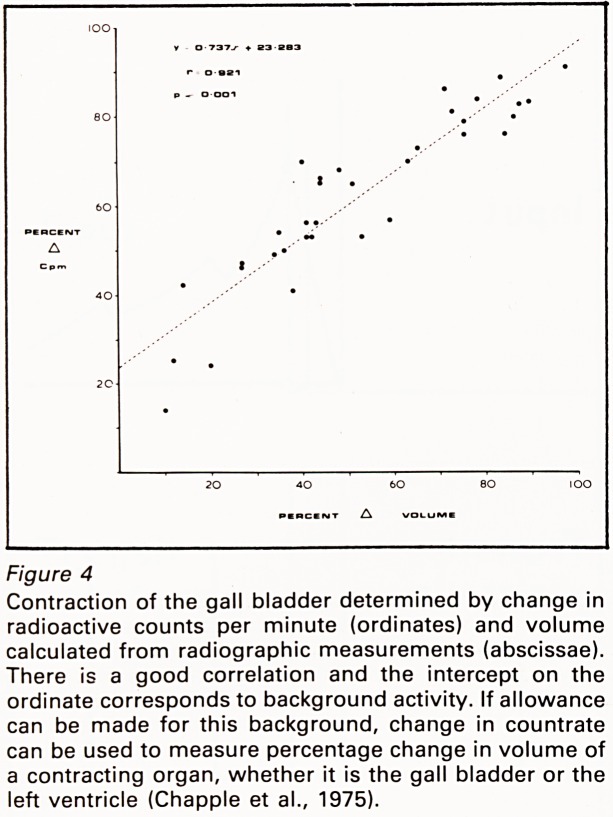


**Figure 5 f5:**
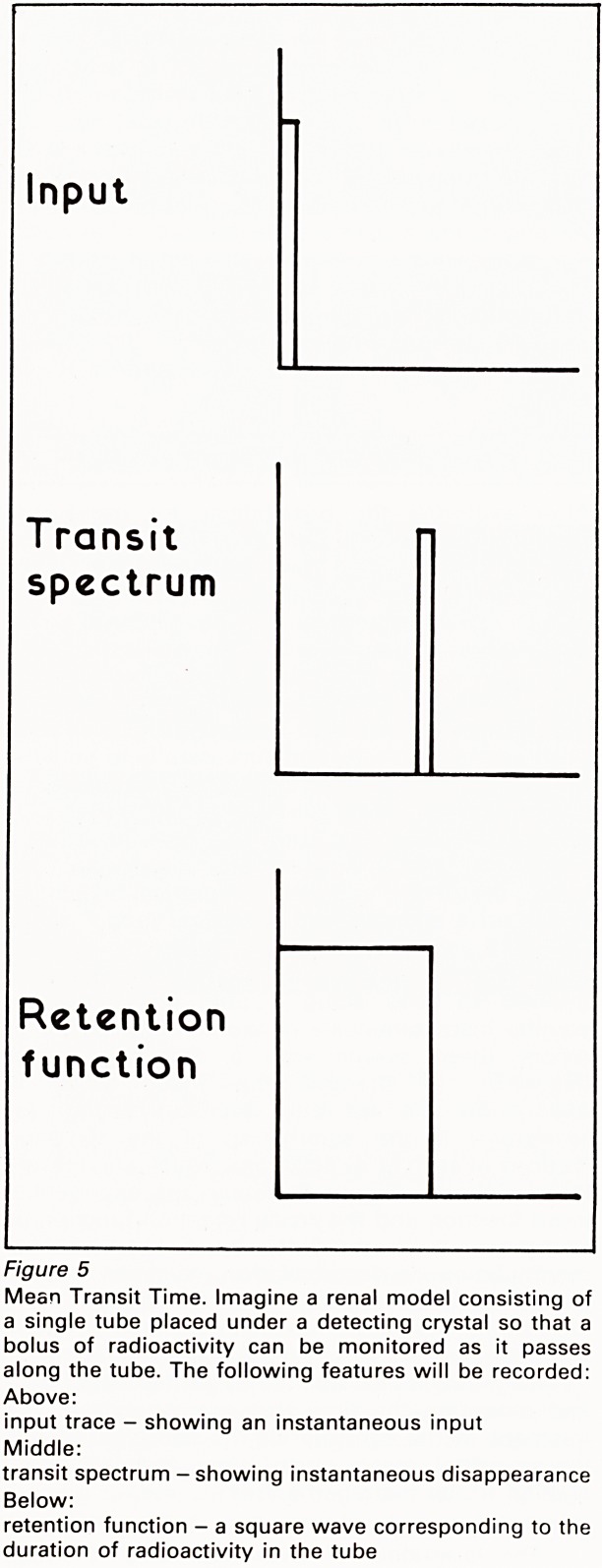


**Figure 6 f6:**
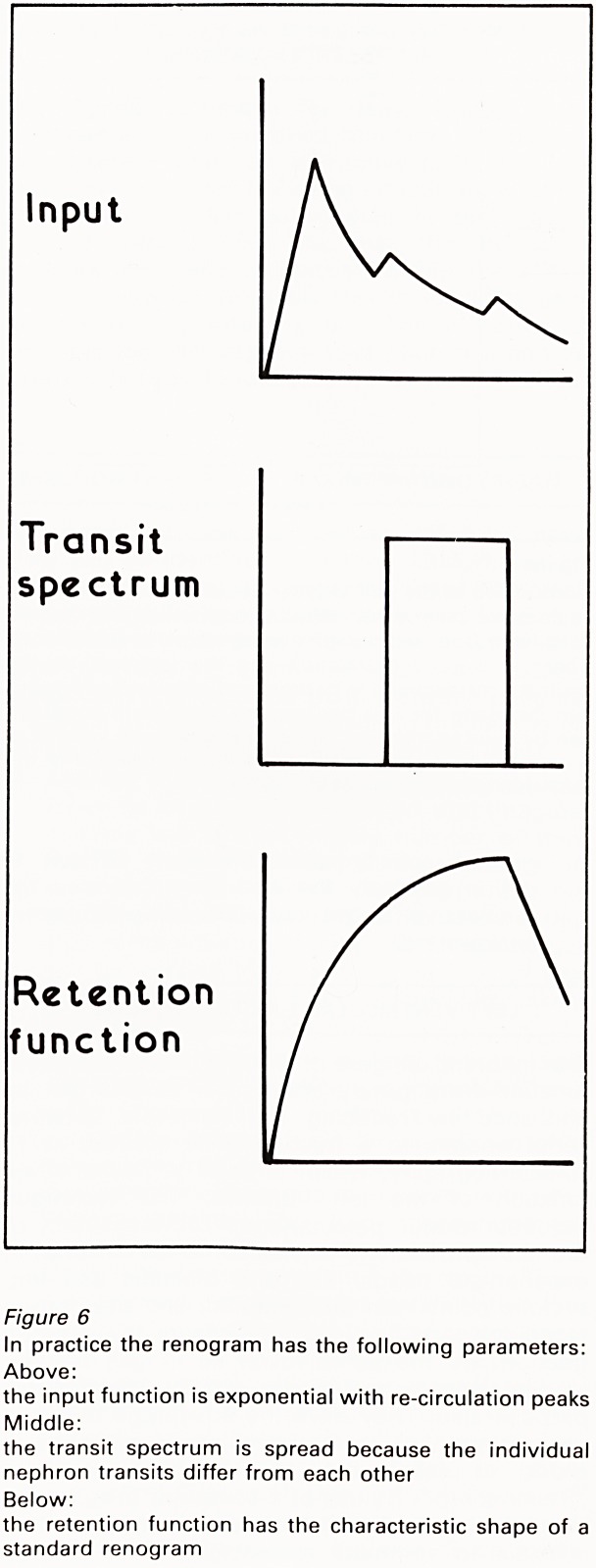


**Figure 7 f7:**
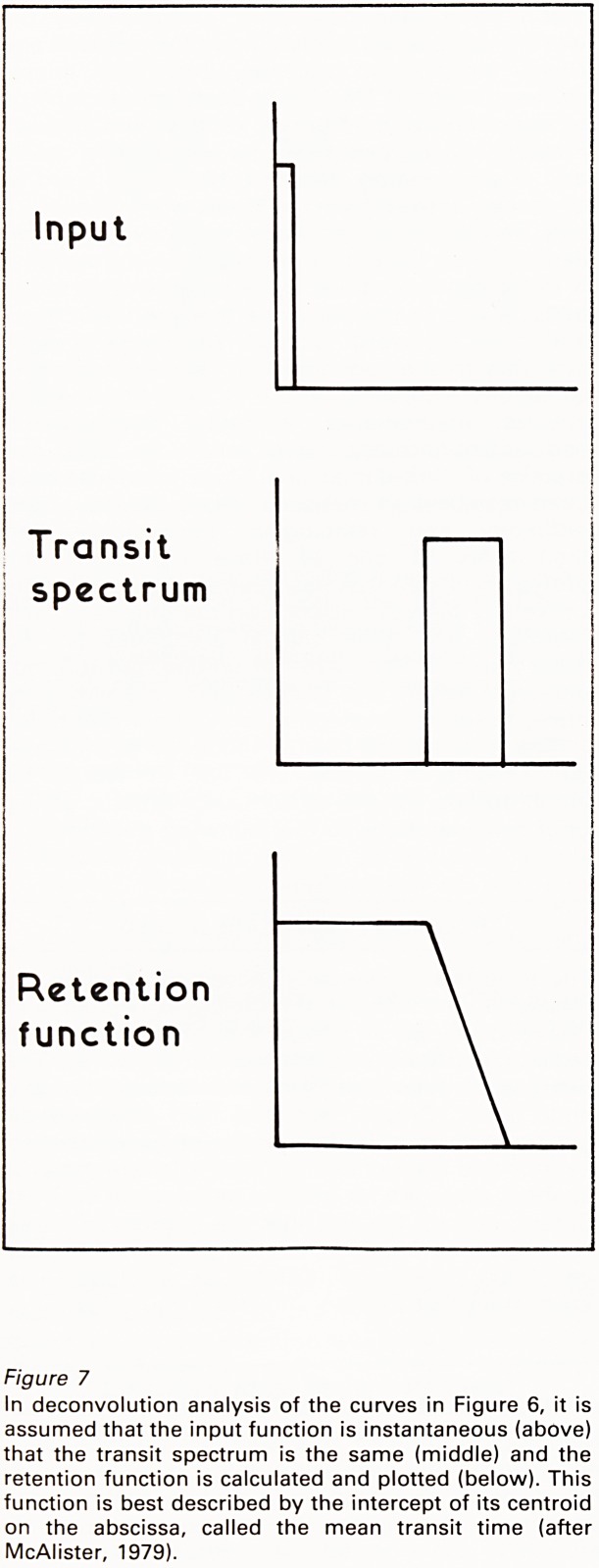


**Figure 8 f8:**
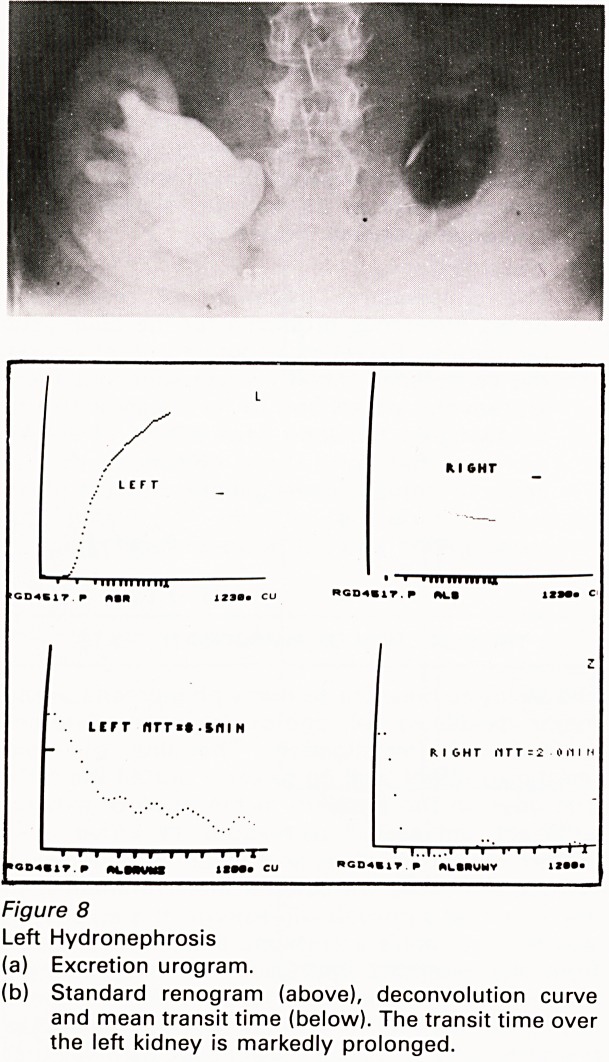


**Figure 9 f9:**